# APOBEC Reporter Systems for Evaluating diNucleotide Editing Levels

**DOI:** 10.1089/crispr.2023.0027

**Published:** 2023-10-10

**Authors:** Amanda E. Rieffer, Yanjun Chen, Daniel J. Salamango, Sofia N. Moraes, Reuben S. Harris

**Affiliations:** ^1^ Department of Biochemistry, Molecular Biology, and Biophysics, University of Minnesota—Twin Cities, Minneapolis, Minnesota, USA; University of Texas Health San Antonio, San Antonio, Texas, USA.; ^2^ Department of Biochemistry and Structural Biology, University of Texas Health San Antonio, San Antonio, Texas, USA; and University of Texas Health San Antonio, San Antonio, Texas, USA.; ^3^ Howard Hughes Medical Institute, University of Texas Health San Antonio, San Antonio, Texas, USA.

## Abstract

Precision genome editing has become a reality with the discovery of base editors. Cytosine base editor (CBE) technologies are improving rapidly but are mostly optimized for TC dinucleotide targets. Here, we report the development and implementation of APOBEC Reporter Systems for Evaluating diNucleotide Editing Levels (ARSENEL) in living cells. The ARSENEL panel is comprised of four constructs that quantitatively report editing of each of the four dinucleotide motifs (AC/CC/GC/TC) through real-time accumulation of eGFP fluorescence. Editing rates of APOBEC3Bctd and AIDΔC CBEs reflect established mechanistic preferences with intrinsic biases to TC and GC, respectively. Twelve different (new and established) base editors are tested here using this system with a full-length APOBEC3B CBE showing the greatest on-target TC specificity and an APOBEC3A construct showing the highest editing efficiency. In addition, ARSENEL enables real-time assessment of natural and synthetic APOBEC inhibitors with the most potent to-date being the large subunit of the Epstein–Barr virus ribonucleotide reductase. These reporters have the potential to play important roles in research and development as precision genome engineering technologies progress toward achieving maximal specificity and efficiency.

## Introduction

Scientists have worked for decades to develop genome engineering technologies safe enough for use in human gene therapy (reviewed by Refs.^1,2^). However, these technologies have been limited by concerns over insertion mutagenesis (integrating retroviruses)^3^ and DNA breakage (zinc finger nucleases, transcription activator-like endonucleases)^4^ and, in all instances, the possibility of off-target mutagenesis and cancer (reviewed by Refs.^1,2^). In addition, these technologies are not ideal for correcting single-nucleotide variants, which are the most common cause of genetic disease in humans.^5,6^ Examples include reversion of point mutations in the *β-globin* gene responsible for β-thalassemia and sickle cell anemia, reversion of an *LMNA* point mutation in Hutchinson–Gilford progeria, correction of splicing defects in spinal muscular atrophy, and introduction of new point mutations to modulate immune responses.^7–12^

Thus, for these and other diseases, there is a significant need for technologies that enable precise genome engineering, and particularly for correcting single-nucleotide mutations in a safe and efficient manner.

**FIG. 1.**
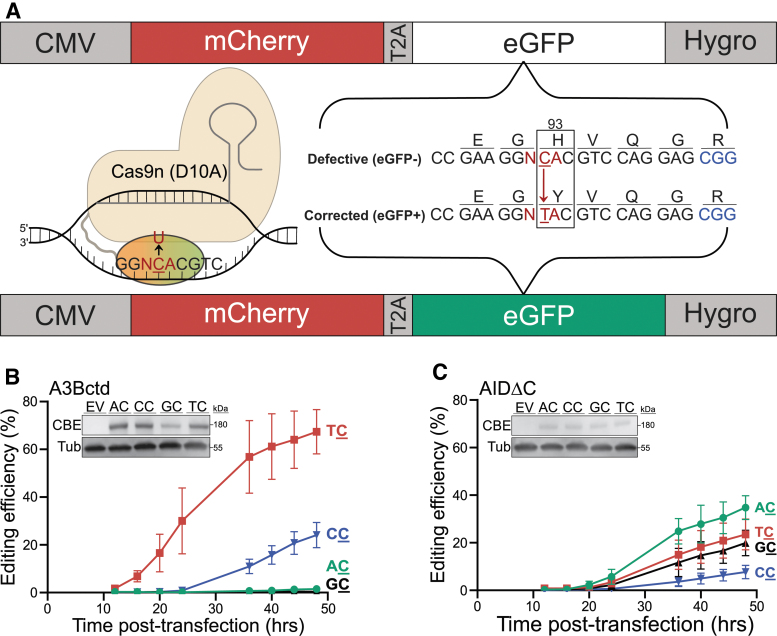
ARSENEL episomally. **(A)** Schematic of ARSENEL. Editing of eGFP His93 (CAC) to Tyr93 (TAC), labeled in red, restores eGFP fluorescence (the target cytosine is underlined, and the PAM site is labeled in blue). Constitutive mCherry expression provides an internal control for quantification of eGFP editing. The inset cartoon depicts editing by a CBE with Cas9n (tan), gRNA (gray), DNA (black), and deaminase (orange/green). **(B, C)** Efficiencies of A3Bctd and AIDΔC CBEs, respectively, using fluorescent microscopy (Cytation imaging) to quantify editing of the four ARSENEL constructs following cotransfection of all reaction components into 293T cells (data points are mean ± SD of biologically independent triplicate experiments). At the final time point, for A3Bctd CBE, all meanwise comparisons are significant (*p* < 0.0001 by two-way ANOVA) except AC versus GC. For AIDΔC CBE, only two meanwise comparisons are significant (AC vs. CC and GC vs. CC with *p* < 0.05 by two-way ANOVA). Inset immunoblots show CBE expression (anti-Cas9) and tubulin as a loading control (different lanes of the same representative immunoblot are shown in **B** and **C** and split only for purposes of presentation). ARSENEL, APOBEC Reporter Systems for Evaluating diNucleotide Editing Levels; CBE, cytosine base editor; CMV, cytomegalovirus; eGFP, enhanced green fluorescent protein; PAM, protospacer adjacent motif; SD, standard deviation.

The feasibility of precision genome engineering improved dramatically with the landmark discovery of efficient cytosine-to-thymine (C-to-T) conversion in human cells using a complex consisting of rat apolipoprotein B mRNA-editing catalytic polypeptide 1 (rApobec1, rA1) linked to a Cas9 nickase (Cas9n) complex (*Streptococcus pyogenes* Cas9-D10A) and bound by an appropriate guide (g)RNA^13^ (e.g., cytosine base editor [CBE] complex shown in Fig. 1A). The complementary base-pairing of the gRNA to a specific DNA target creates a short single-stranded (ss)DNA region (an R-loop), which becomes a substrate for deamination by the covalently linked rA1 enzyme. Cytosine-to-uracil (C-to-U) editing events within the exposed ssDNA region can then become immortalized as C-to-T mutations due to the Cas9n-catalyzed ssDNA break, which directs repair enzymes to the opposing DNA strand (the G-containing strand).^13,14^

Uracil persistence and overall editing efficiencies are further improved by linking the rA1-Cas9n complex to a phage-derived uracil DNA glycosylase inhibitor (Ugi) to prevent uracil excision and error-free repair. This design—rA1-Cas9n/gRNA-Ugi—is known popularly as base editor 3 (BE3).^13^ Since this discovery, a multitude of CBEs have been created by fusing hundreds of different APOBEC family enzymes (wild-type proteins and mutants) to different Cas9 nickase complexes (also wild-type and mutants), as well as varying copy numbers of Ugi.^13,15–19^ Several of these published constructs are used here including BE3,^13^ BE4max (codon optimized rA1-Cas9n-Ugi-Ugi),^19^ APOBEC3B (A3B) C-terminal domain (ctd)-Cas9n-Ugi,^17,18^ full-length A3B-Cas9n-Ugi,^16–18^ activation-induced cytidine deaminase (AID)ΔC-Cas9n-Ugi,^20–22^ and APOBEC3A-N57G-Cas9n-Ugi,^8^ as well as new constructs such as derivatives of A3Bctd-BE3/BE4max.

Despite thousands of constructs, hundreds of thousands of editing reactions, and millions of quantified edits, CBEs still have significant room for improvement.^1,2^ The most challenging issue is achieving the “right balance” between on-target specificity (the ability of a CBE to specifically deaminate a single-target cytosine and not bystander or off-target cytosines) and editing efficiency (the rate at which the on-target cytosine is edited). Efficient base editors such as BE3, BE4max, and A3A invariably catalyze off-target editing events proximal to the target cytosine (here called bystander edits) and gRNA-dependent and -independent events throughout the genome of cells.^23–25^ Conversely, a few editors have been engineered to exhibit higher degrees of on-target specificity, such as YEE-BE4max and eA3A, but an increase in specificity often comes at the cost of a decrease in editing efficiency.^8,24^

Two decades of research have shown that the −1 nucleobase directly upstream of the target cytosine has the largest effect on cytosine deaminase substrate specificity because it makes direct intermolecular contacts with the editing enzyme.^26–35^ Thus, most CBEs show a strong bias for TC dinucleotide motifs, which reflects their natural substrate preference, including rA1 (plus many engineered variant derivatives), A3A, A3B, A3Bctd, and Anc689 complexes.^8,13,16–19^ Therefore, the intrinsic −1 nucleobase preference further constrains CBE utility by making it harder to develop editors for other dinucleotide substrate motifs.

Here, we describe a set of four reporters that can be used episomally or chromosomally to compare CBE dinucleotide specificities and editing efficiencies in real time. These APOBEC Reporter Systems for Evaluating diNucleotide Editing Levels (ARSENEL) have four key elements. First, a single C-to-U edit (C-to-T mutation) converts a CAC codon (His93) into a TAC codon (Tyr93), which restores eGFP function and enables quantification by fluorescence imaging or flow cytometry. Second and unique to our studies here, this target codon is preceded by a Gly92 codon, which has a third base wobble enabling A, C, G, or T to be placed immediately upstream of the target cytosine in the first position of codon 93. Third, an *mCherry* reporter is linked using a T2A “self-cleaving” (ribosome skipping) motif to the downstream *eGFP* reporter, which provides a robust internal control for editing quantification.

Last, the ARSENEL system is comprised of four different plasmid constructs that can be separately transfected into cells for direct episomal editing reactions or used to produce VSV-pseudotyped lentiviral supernatants for transduction into almost any cell line and chromosomal DNA editing reactions, thus mimicking many different human genomic DNA disease alleles that can be corrected by precise single C-to-T mutations. The ARSENEL system is used here to evaluate the dinucleotide preferences of a variety of CBEs. In addition, the ARSENEL system can provide a quantitative readout of regulators of CBE activity including deaminase inhibitors.

## Materials and Methods

### Reporter constructs

All plasmids used in this study are available through Addgene (MA), and oligonucleotide sequences are listed in Supplementary Table S1. The TC dinucleotide reporter [pLenti-CMV-mCherry-T2A-eGFP-Gly92(GGT)-His93(CAC)-Hygro] has been published (No. 198882; Addgene).^17,36^ The Y93 AC, CC, and GC dinucleotide reporters were created by site-directed mutagenesis of the TC construct (constructs available through Nos. 198883, 198885, and 198884, respectively; Addgene). gRNAs for editing of each of the four reporters were ordered as complementary oligonucleotides and cloned using Golden Gate procedures^37^ into an optimized MLM3636^17^ (No. 43860; Addgene, deposited by K. Joung lab). All reporter constructs were confirmed by Sanger sequencing (Azenta/Genewiz, NJ) or whole-plasmid sequencing (Plasmidsaurus, OR).

### CBE constructs

Human A3A (No. 109425; Addgene),^17^ human eA3A,^8^ human full-length A3B (No. 198889; Addgene),^17^ human A3Bctd (No. 109426; Addgene),^17^ human AIDΔC CBE (No. 198890; Addgene),^36^ and rat A1 (No. 73021; Addgene, deposited by D. Liu lab)^13^ CBEs have been reported. A3Bctd-L7G was PCR subcloned from an existing construct^38^ and used to replace rA1 in BE3 by restriction digestion and ligation (No. 198886; Addgene). A3Bctd-D314E (No. 198887; Addgene) and eA3A (No. 207165; Addgene) were created using site-directed mutagenesis. The BE4max plasmid was a gift from the B. Moriarity laboratory (No. 112093; Addgene, deposited by D. Liu lab).^19^ The BE4max plasmid was reconstructed by removing the deaminase and inserting Esp3I sites 5′ from the Cas9 gene (No. 198891; Addgene). Deaminase open reading frames were generated by PCR and cloned into the reconstructed BE4max backbone via Esp3I Golden Gate cloning^37^ (A3Bctd-max, No. 198888; Addgene, A3Bmax, No. 207167; Addgene, eA3Amax, No. 207166; Addgene).

BORF2 constructs have been reported.^39,40^ All CBE constructs were confirmed by Sanger sequencing (Azenta/Genewiz) or whole-plasmid sequencing (Plasmidsaurus). WT constructs match the following GenBank accessions: *Homo sapiens* A3B (NM_004900), *H. sapiens* A3A (NM_145699), *H. sapiens* AID (NM_020661.4), *Rattus norvegicus* APOBEC1 (NC_051339.1), *S. pyogenes* Cas9 (WP_038431314.1), and Epstein-Barr virus (EBV) BORF2 (V01555.2).

### Episomal base editing experiments

Semiconfluent 293T cells in three 24-well plates were transfected (in parallel) with 50 ng gRNA, 100 ng reporter, and 150 ng of each base editor (25 min at RT with 0.9 μL of TransIT LT1 [Mirus] and 50 μL of serum-free RPMI). One plate was imaged using a Cytation1 Multi-Mode Reader (Agilent, CA) as described^36^ over a 48-h period (configured with Texas Red [586/647] and GFP [469/525]). After 48 h of incubation, the second and third plates were harvested for immunoblots and flow cytometry. For flow cytometry analysis, trypsinized and PBS-washed cells were placed in a 96-well round-bottom plate. A minimum of 20,000 events were acquired for each condition using a BD LSRFortessa or a BD FACSCanto flow cytometer (BD, OR). Here, percent editing was calculated by dividing the number of eGFP and mCherry double-positive cells by the total number of mCherry-positive cells and multiplying by 100 (FlowJo software; BD).

### Chromosomal base editing experiments

Semiconfluent six-well plates of 293T cells were transfected with 500 ng of an HIV-1 Gag-Pol packaging plasmid and of a VSV-G expression plasmid cocktail (a 2:1 ng ratio), and 500 ng of each base editing reporter. Viruses were harvested 48 h post-transfection and used to transduce target cells (multiplicity of infection, MOI = 0.3). Forty-eight hours post-transduction cells were subjected to hygromycin selection at a 400 μM concentration. After selection, >95% of cells were mCherry positive by flow cytometry. Transduced, mCherry-positive cells were transfected with 425 ng of CBE and 75 ng of targeting gRNA into four parallel semiconfluent 24-well plates. As with the episomal experiments above, one plate was imaged using a Cytation or Incucyte instrument over a 72-h period.

The other three plates prepared in parallel were harvested after 72 h of incubation for editing quantification by flow cytometry as explained in the previous section, genomic DNA isolation for reporter target analysis, and for expression confirmation immunoblotting.

### Live-cell imaging analyses

**FIG. 2.**
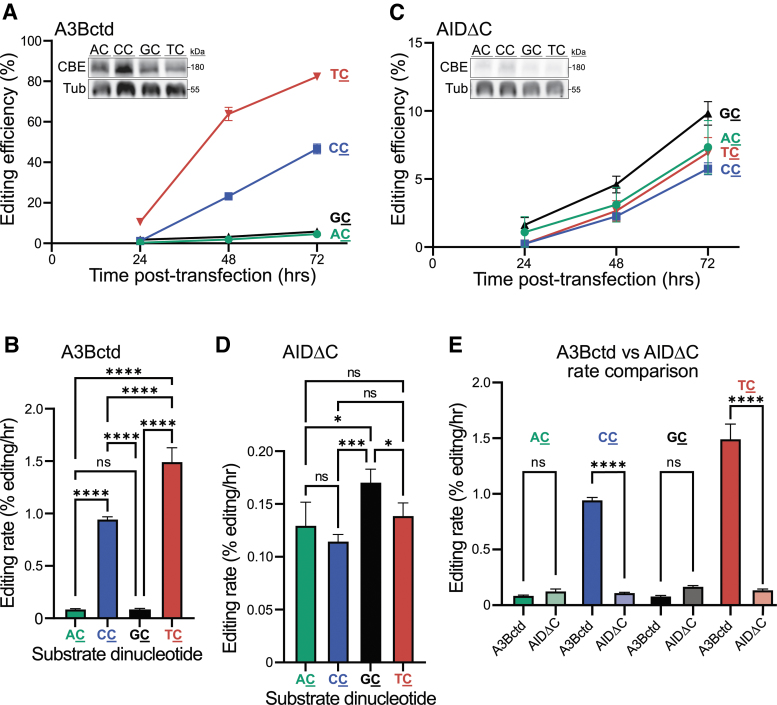
ARSENEL chromosomally. **(A, C)** Efficiencies of A3Bctd and AIDΔC CBEs, respectively, using fluorescent microscopy (Cytation imaging) to quantify editing of the four chromosomally integrated ARSENEL constructs following cotransfection of CBE and gRNA plasmids into 293T cells (data points are mean ± SD of biologically independent triplicate experiments; most error bars are smaller than the symbols). At the final time point, for A3Bctd CBE, all meanwise comparisons are significant (*p* < 0.0001 by two-way ANOVA) except AC versus GC. For AIDΔC CBE, only two meanwise comparisons are significant (GC vs. TC and GC vs. CC with *p* < 0.01 by two-way ANOVA). Inset immunoblots show CBE expression (anti-Cas9) and tubulin as a loading control (different lanes of the same representative immunoblot are shown in **A** and **C** and split only for purposes of presentation). **(B**, **D)** Editing rates from results in **A** and **C** after linear regression calculations (ns = not significant; *p < 0.05; ***p < 0.001; ****p < 0.0001 by ordinary one-way ANOVA). **(E)** A3Bctd CBE and AIDΔC CBE editing rate comparisons after linear regression calculations compiled from **B, D** (ns = not significant; ****p < 0.0001 by two-way ANOVA).

The Cytation1 Multi-Mode Reader (Agilent) configured with Texas Red (586/647) and GFP (469/525) light cubes was used to image and analyze data for Figures 1, 2, and 4. Live-cell images were captured using a 4 × objective at several time points after the initial transfection (e.g., Supplementary Fig. S1A). Temperature was maintained at 37°C for the duration of imaging. Nine images were taken per well in a fixed 3 × 3 grid. After background subtraction, a dual-mask analysis was used to quantify edited versus nonedited cells. All mCherry-positive cells were first identified as the primary mask using the fluorescence threshold as well as size exclusion. Next, a secondary mask was created within the primary mask to identify eGFP-positive cells using a GFP fluorescence threshold. The ratio of double-positive cells to mCherry-positive cells was then used to calculate relative editing frequencies over time.

Data for Figure 5 were collected and analyzed using an Incucyte (Sartorius, Germany). Live-cell images of orange, green, and phase image channels were captured with a 4 × objective every 4 h after the initial transfection with five images per well in a fixed grid. mCherry- and GFP-positive cells were identified with internal cellular analysis software.

### Editing quantification by Sanger sequencing

After sample collection, genomic DNA was isolated (Qiagen Gentra Puregene). A 712 bp segment of the eGFP target region was amplified by high-fidelity PCR using Phusion polymerase. PCR products were gel purified and submitted for Sanger sequencing by Azenta/Genewiz, ACGT (MD), or Eurofins (TX). Chromatograms received from Sanger sequencing were uploaded to EditR for peak quantification.^41^ For individual clone analysis, genomic DNA was prepared and the target editing region PCR amplified as described above, and then inserted into pJET1.2/blunt Cloning Vector using the CloneJET PCR Cloning Kit (Thermo Fisher Scientific, MA). Individual colonies were picked after transformation into *Escherichia coli*, miniprepped, and then submitted for Sanger sequencing.

### Immunoblotting

Cells were collected and lysed in 2 × reducing sample buffer, and then incubated at 98°C for 15 min. All samples collected from an individual experiment were run on the same gel to compare protein expression levels as accurately as possible. Samples were separated by a Criterion 4–20% gel (No. 5671095; Bio-Rad), and then transferred to PVDF membranes. Antibody incubation was conducted as previously reported.^42^ Primary antibodies used include rabbit anti-Cas9 (No. ab189380, 1:5000; Abcam), mouse anti-tubulin (No. T5168, 1:10,000; Sigma-Aldrich), and rabbit anti-FLAG (No. F7425, 1:5000; Sigma-Aldrich). Secondary antibodies used were goat anti-rabbit IRdye800 (No. 925-32211, 1:10,000; LI-COR) and goat anti-mouse IRdye680 (No. 926-69020, 1:10,000; LI-COR).

### Statistical analyses

GraphPad Prism 9.0 was used for statistical analyses of quantitative data. Sample size, biologically independent experiments, and statistical analyses are given in the figure legends. Unless otherwise stated in the legends, error bars represent one standard deviation from the mean, and two-way ANOVA tests were used to assess significance (ns = not significant; *p < 0.05; **p < 0.01; ***p < 0.001; ****p < 0.0001).

## Results

### Episomal ARSENEL

We previously developed eGFP-based reporters for quantification of APOBEC-catalyzed base editing in living cells.^17,36^ One of these constructs relies on editing of a mutant CAC codon (His93) to a TAC codon (Tyr93) (reporter schematic in Fig. 1A includes a depiction of a CBE complex). This single base change restores eGFP fluorescence and, together with an upstream constitutively expressed mCherry internal control, enables robust quantification of editing frequencies in living cells using fluorescent imaging or flow cytometry [i.e., editing efficiency (%) = (total number of eGFP-positive cells/total number of mCherry-positive cells) × 100] (graphical schematic of workflow in Supplementary Fig. S1A). In addition, this dual-fluorescent reporter construct can be transiently transfected or stably transduced into any cell line to evaluate efficiencies of episomal and chromosomal editing, respectively.

Here, we report the creation of an isogenic set of four constructs in which the wobble position of the Gly92 codon (GGT) immediately upstream of the His93 codon (CAC) is engineered to reflect the four possible dinucleotide editing combinations. Specifically, in addition to the existing GGT construct, the Gly92 codon has been changed by site-directed mutagenesis into a GGA, GGC, and GGG (represented by N in Fig. 1A). In all instances, the encoded amino acid is still glycine, but this set of four “wobble” constructs now enables systematic comparison of the intrinsic dinucleotide editing preferences of a variety of different CBEs. Accordingly, the corresponding gRNAs are also changed by one nucleobase to maintain perfect complementarity, but the target cytosine, the protospacer-adjacent motif (PAM), and all other reaction components are identical.

To test whether these constructs accurately report known intrinsic deaminase preferences, we first compared the dinucleotide editing preferences of an A3Bctd CBE and an AIDΔC CBE in transient transfection experiments using 293T cells. The intrinsic preference of A3Bctd is TC and AID is RC (R = A or G).^14,33,43–48^ The TC reporter is edited efficiently by A3Bctd CBE (Fig. 1B), in agreement with our original studies.^36^ Fluorescent signal (eGFP editing) from on-target editing is first detectable 12–16 h post-transfection, and it reaches an average of 67% editing by 48 h. This maximal editing frequency likely reflects a balance of true on-target editing and bystander events that can inactivate eGFP (Supplementary Fig. S1B), as well as random fluctuations in delivering three plasmids to cells by cotransfection. The CC reporter is also edited by the A3Bctd CBE, but efficiencies are lower with a maximum of 24% editing after 48 h.

As anticipated, AC and GC dinucleotide substrates are strongly disfavored by A3Bctd^26,43,46,47^ with <1% editing through the 48-h experimental duration. Parallel reactions collected at 40 h post-transfection confirm CBE expression (immunoblot inset in Fig. 1B) and enable independent confirmation by flow cytometry (Supplementary Fig. S1C).

In comparison, the AIDΔC CBE exhibits lower overall editing efficiencies in transient reactions and prefers AC with 35% editing after 48 h (Fig. 1C). The AIDΔC CBE also shows significant activity with the GC reporter (20%) and, unexpectedly, high activity with the TC reporter (23%) 48 h after transfection. The slight deviation from the reported RC preference of wild-type AID for purine-C dinucleotide substrates is not due to different expression levels between each editing reaction (inset immunoblot in Fig. 1C) but may be due to a suboptimal −2 nucleobase in the reporter and/or to constraints imposed by direct fusion of AIDΔC to the Cas9n-Ugi complex.^32,33,44,48^ Flow cytometry analysis did not show a clear dinucleotide preference for the AIDΔC CBE, which has also been seen previously^20,22^ and may be due to the elevated sensitivity of flow cytometry to pick up cells with positive, although low, eGFP fluorescence (Supplementary Fig. S1D).

Nevertheless, taken together, the results with the A3Bctd and AIDΔC CBE constructs combine to show that the ARSENEL system enables an assessment of dinucleotide editing efficiencies of CBEs in real time in living cells.

### Chromosomal ARSENEL reduces experimental variability and reflects intrinsic CBE dinucleotide preferences

Next, retroviral transduction was used to introduce each reporter construct stably into the chromosomal DNA of 293T cells (MOI = 0.3). This pool-based approach ensures that most cells have only one integrated reporter and simultaneously minimizes integration position effects common for single clones. Thus, results with chromosomal ARSENEL are anticipated to provide a less biased population-based readout of the efficiency and specificity of CBE editing of single-genomic cytosine nucleobases in each of the four dinucleotide contexts. Using this system, the A3Bctd CBE exhibits a strong dinucleotide specificity for TC substrates with 82% editing at 72 h post-transfection (Fig. 2A). The next best substrate is CC with 47% at 72 h, followed distantly by GC and AC with 5.8% and 4.6%, respectively, at 72 h.

Flow cytometry data confirm high frequencies of correction of the TC reporter followed by the CC reporter, and negligible editing of both the AC and GC reporters (Supplementary Fig. S2A). If the slopes for each of the reactions in Figure 2A are calculated, the rates of editing can be directly compared for each of the four substrates as an additional quantitative measure of efficiency. Here, the A3Bctd CBE edits TC at 1.5% per hour, CC at 0.9% per hour, and AC or GC at <0.1% per hour (Fig. 2B). The differences in these editing rates are most likely due to the intrinsic preferences of the deaminase component of each otherwise identical CBE complex as expression levels are similar for the most (TC) and least (GC) efficient editing reactions (immunoblots in inset of Fig. 2A).

A different picture emerges from AIDΔC CBE chromosomal ARSENEL experiments. First, in comparison with the episomal editing data described above, a significant drop in editing efficiency is observed even for the best substrate, GC, which only reaches 9.8% by 72 h (Fig. 2C). This is consistent with prior biochemical observations that AID is >10-fold less active than A3Bctd *in vitro* using recombinant proteins and oligonucleotide substrates.^49,50^ Second, AIDΔC CBE prefers GC over AC, TC, and CC using fluorescent plate readings (Fig. 2C), although parallel reactions analyzed by flow cytometry were less clear likely due to elevated background fluorescence (Supplementary Fig. S2B). The plate reader results can also be converted to editing rates by calculating the slopes of each time course experiment, with GC prevailing as the preferred substrate with 0.17% editing per hour (Fig. 2D).

The use of editing rates (percent editing per hour) enables cross-comparisons of the dinucleotide preferences of different CBE constructs within the same chromosomal editing experiment. For example, the A3Bctd CBE significantly outperforms the AIDΔC CBE on the TC and CC target dinucleotides and has indistinguishably low editing rates for the AC or GC sites (Fig. 2E).

### Sanger sequencing of chromosomal reporter pools enables quantification of on-target and bystander editing efficiencies

**FIG. 3.**
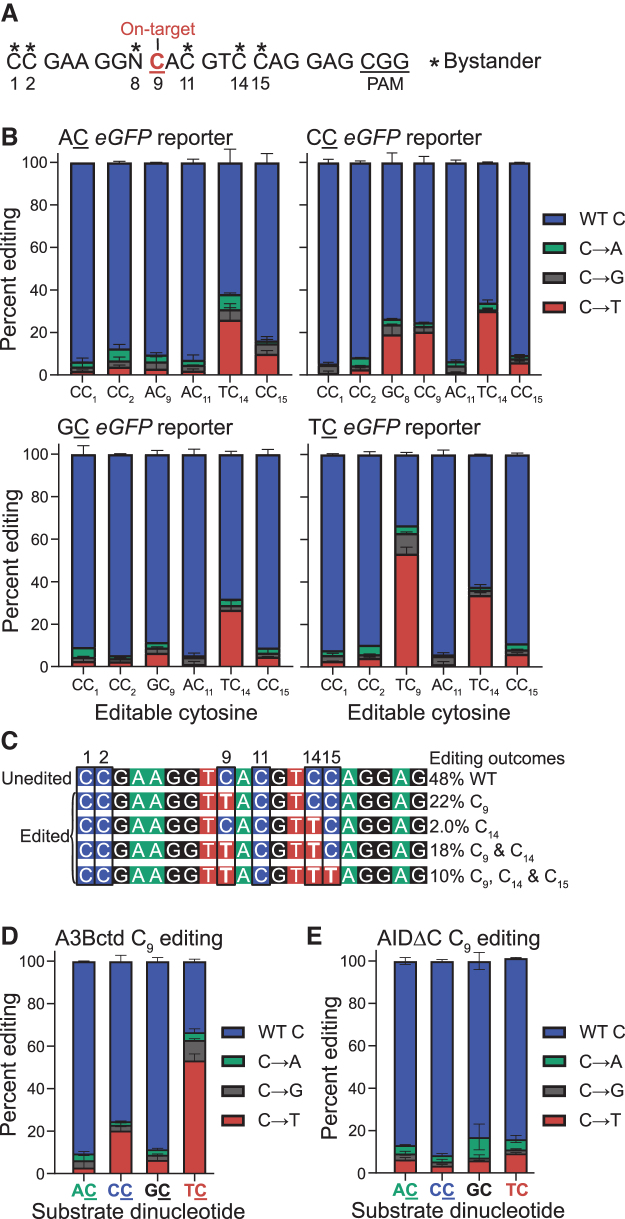
On-target and bystander editing events in chromosomal editing reactions with ARSENEL system. **(A)** Schematic of the gRNA-displaced region in the ARSENEL constructs. The on-target cytosine (C_9_) is highlighted in red and bystander cytosines are marked by asterisks. The CGG PAM site is underlined. **(B)** Stacking bar graph representations of A3Bctd CBE on-target and bystander editing events using each of the four different ARSENEL constructs from direct Sanger sequencing of PCR amplicons derived from genomic DNA. Unedited (WT) cytosines are shaded blue, C-to-A edits shaded green, C-to-G edits shaded gray, and C-to-T edits shaded red. Error bars represent the differences between the means of two biologically independent replicates. **(C)** Editing allele frequencies and linkage analysis for individually cloned and Sanger-sequenced PCR products from the gRNA-targeted region of eGFP from A3Bctd CBE editing reactions (percentages determined from *n* = 50 independent sequences). No indel events were detected in 50 independent clones. **(D, E)** Stacking bar graphs of on-target C_9_ editing by A3Bctd CBE and AIDΔC CBE, respectively, from direct Sanger sequencing of PCR amplicons derived from genomic DNA. Unedited cytosines are shaded blue, C-to-A edits shaded green, C-to-G edits shaded gray, and C-to-T edits shaded red. Error bars represent the differences between the means of two biologically independent replicates.

An additional property of the ARSENEL system is that the gRNA binding region in chromosomally transduced pools with one reporter per cell can be recovered easily in bulk by high-fidelity PCR and then Sanger-sequenced to assess frequencies of on-target specificity for the target cytosine and bystander editing (in contrast, editing rates of a transiently transfected reporter cannot be quantified in this manner because wild-type reporter copy numbers can far exceed one per cell and the vast majority of targets are not edited). The gRNA binding window has a total of six cytosines in three different dinucleotide contexts that can be edited by a CBE (numbered 1, 2, 9, 11, 14, and 15 in Fig. 3A).

C-to-T editing at C_9_ restores eGFP function, whereas editing at the majority of the other cytosines is expected to be aphenotypic, with the exception of a TAG stop codon produced by C_15_-to-T editing events (Supplementary Fig. S1B). Thus, Sanger sequencing data, analyzed using the chromatogram peak quantification program EditR,^41^ can provide a simultaneous assessment of both on-target C_9_ and bystander cytosine editing frequencies.

As anticipated from eGFP fluorescence data above, an on-target edit of TC_9_-to-TT by the A3Bctd CBE is present in 53% of sequences after 72 h of incubation (bottom right graph in Fig. 3B). Surprisingly, TC_14_ is also edited in more than 30% of these sequences (bottom right graph in Fig. 3B). In other words, more than half of the on-target C_9_ editing events are accompanied by a bystander event at TC_14_, which is likely to be phenotypically silent due to its position in the wobble position of the Val94 codon (Supplementary Fig. S1B).

To investigate coincident editing events at C_9_ and C_14_ and other cytosine positions within the gRNA region, genomic DNA collected from the A3Bctd-edited TC reporter cells was PCR amplified and cloned into a pJet vector creating a pool of plasmids with differentially edited eGFP sequences. After transformation into *E. coli*, plasmid DNA from independent single colonies was miniprepped and Sanger sequenced (*n* = 50). Results from these individual clone sequences show that 22% of clones exhibit exclusive on-target TC_9_ editing events, 18% show both TC_9_ and TC_14_ editing events, and only 2% have exclusive TC_14_ editing events (Fig. 3C). Because TC_14_ is frequently edited in combination with C_9_, we hypothesize that editing of the primary target TC_9_ occurs before editing of C_14_.

Interestingly, this individual clone analysis also reveals that 10% of the sequences have an identical combination of multiple edited sites (C_9_, C_14_, and C_15_) and likely represent hyper-edited targets incapable of encoding a functional eGFP protein (due to C_15_-to-T creating a stop codon). No insertion–deletion (indel) mutations were recovered using this approach suggesting that such outcomes may only comprise a small minority of all A3Bctd CBE editing events.

Notably, the A3Bctd CBE exhibits >25% editing at TC_14_ in all four dinucleotide reporters, further underscoring the TC preference of this construct and the silent nature of the TC_14_-to-TT mutation (Fig. 3B). However, this unexpected bystander outcome has two potential benefits. First, the phenotypically silent TC_14_-to-TT editing site provides an unbiased metric for bystander editing versus on-target TC_9_ editing. Second, it provides an internal comparison for studies seeking to change the dinucleotide specificity of an originally TC-preferring CBE (e.g., A3Bctd CBE below). In contrast, levels of all other C-to-T edits at the other cytosine positions in the four different reporters are substantially lower or negligible, as are transversion C-to-A and C-to-G outcomes (Fig. 3B). The only statistically significant transversion editing outcomes are 9.6% TC_9_-to-TG in the TC reporter and 7.1% TC_14_-to-TA in the AC reporter.

However, such noncanonical outcomes are not detected in 50 individual clone Sanger sequence reads (Fig. 3C). The Sanger sequencing results also suggest that the duplex DNA adjacent to the gRNA-binding region may be protected from editing as no mutations in flanking regions are detected.

Bar graphs afford another way to summarize and compare PCR product sequencing data for ARSENEL editing outcomes at the C_9_ position (Fig. 3D, E). Editing by A3Bctd CBE leads preferentially to C-to-T mutations at TC_9_ (53%) over CC_9_ (20%), GC_9_ (6.5%), and AC_9_ (3.9%) (Fig. 3D). These percentages and relative differences between the different dinucleotide targets are similar to the phenotypic (eGFP-positive) results described above for real-time editing of the four different ARSENEL reporters in a chromosomal DNA context (Fig. 2A, B, and Supplementary Fig. S2A). In comparison, the PCR sequencing results of chromosomal editing by the AIDΔC CBE show that the frequency of C_9_-to-T mutations largely parallel functional results described above (Fig. 2C, D, and Supplementary Fig. S2B) with ∼5–10% C-to-T edits after 72 h for each dinucleotide target (Fig. 3E).

### Loop 7 changes alter the dinucleotide editing preferences of A3Bctd CBE

**FIG. 4.**
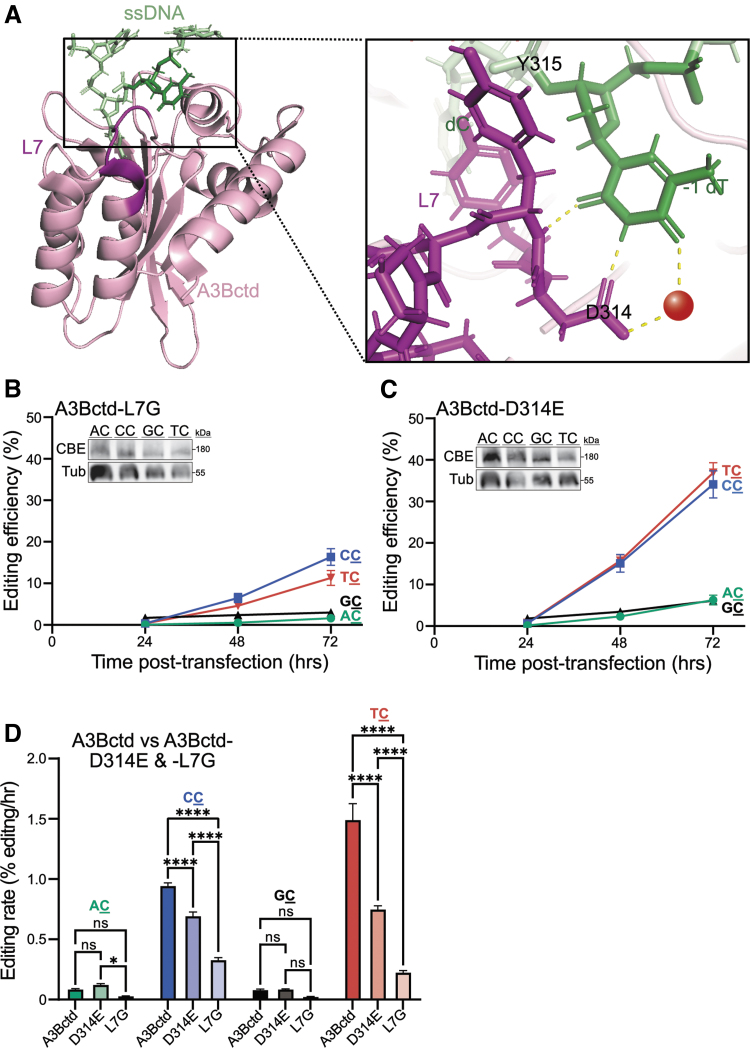
A3Bctd loop 7 dictates substrate specificity and editing efficiency. **(A)** Ribbon schematic of A3Bctd (pink) bound to ssDNA (green) with loop 7 residues highlighted in magenta (PDB: 5TD5). Three H-bonds govern the selection of TC substrates including one mediated by water (red). **(B, C)** Efficiencies of A3Bctd-L7G and A3Bctd-D314E CBEs, respectively, using fluorescent microscopy (Cytation imaging) to quantify editing of the four chromosomally integrated ARSENEL constructs following cotransfection of CBE and gRNA plasmids into 293T cells (data are mean ± SD of biologically independent duplicate experiments, each with two technical replicates). For the L7G construct, the 72-h editing time point is significantly different for CC versus TC (*p* < 0.05) and, for the D314E construct, the same time point is not significant for CC versus TC (*p* > 0.05 by two-way ANOVA). Different lanes of the same representative immunoblot from reactions 72 h post-transfection are shown in **(B, C)** and split only for purposes of presentation. (**D**) Bar graphs comparing the editing rates for A3Bctd, A3Bctd-L7G, and A3Bctd-D314E CBEs after linear regression calculations (data from Fig. 2A–C; ns = not significant; *p < 0.05; ****p < 0.0001 by two-way ANOVA).

To further investigate the utility of the ARSENEL system, we asked whether structure-guided changes to the loop 7 region of A3Bctd can alter dinucleotide editing preferences in the same manner as reported for recombinant proteins *in vitro*.^26^ Crystal structures of A3Bctd and ssDNA-A3Bctd have demonstrated that the target deoxy-cytosine nucleotide is deep within the active site pocket and the upstream deoxy-thymine nucleotide (−1 T) is engaged by three distinct hydrogen (H)-bonds (two directly with Asp314, one water-mediated; Fig. 4A).^26,28,35,51^ Structural and biochemical studies have shown that swapping the entire loop 7 region of A3Bctd with the corresponding region from A3Gctd changes the intrinsic dinucleotide preference from TC to CC.^26,35^

Moreover, a single amino acid substitution in A3Bctd loop 7, Asp314-to-Glu, extends the amino acid side chain by one carbon and is hypothesized to break the H-bond network with the −1 T and create a more favorable H-bond environment for accommodation of a −1 C.^35^ Thus, these two changes to loop 7 (full loop swap and Asp314-to-Glu) are expected to change the dinucleotide editing preference of the A3Bctd CBE from TC to CC.

This mechanistic connection between loop 7 and dinucleotide preference was tested using chromosomal ARSENEL. In comparison with the parental A3Bctd CBE construct, which has a clear preference for TC over CC substrates, the A3Bctd-L7G and A3Bctd-Asp314-to-Glu CBEs appear to exhibit similar preferences for both the CC and TC reporters (compare data in Fig. 4B, C). In comparison with A3Bctd CBE, where a TC preference predominates (Fig. 2B), the A3Bctd-L7G CBE has a lower overall editing efficiency with a slight CC bias, and the A3Bctd-Asp314-to-Glu CBE has a higher overall editing rate compared with the A3Bctd-L7G CBE, with no significant difference between editing TC and CC chromosomal ARSENEL substrates (Fig. 4D), which is recapitulated in flow cytometry results (Supplementary Fig. S2C, D).

Taken together, these results combine to indicate that loop 7 changes (full loop swap or Asp314-to-Glu) are able to diminish the intrinsic preference of A3Bctd for TC and simultaneously increase it for CC.

### High-efficiency CBEs exhibit elevated bystander editing events

**FIG. 5.**
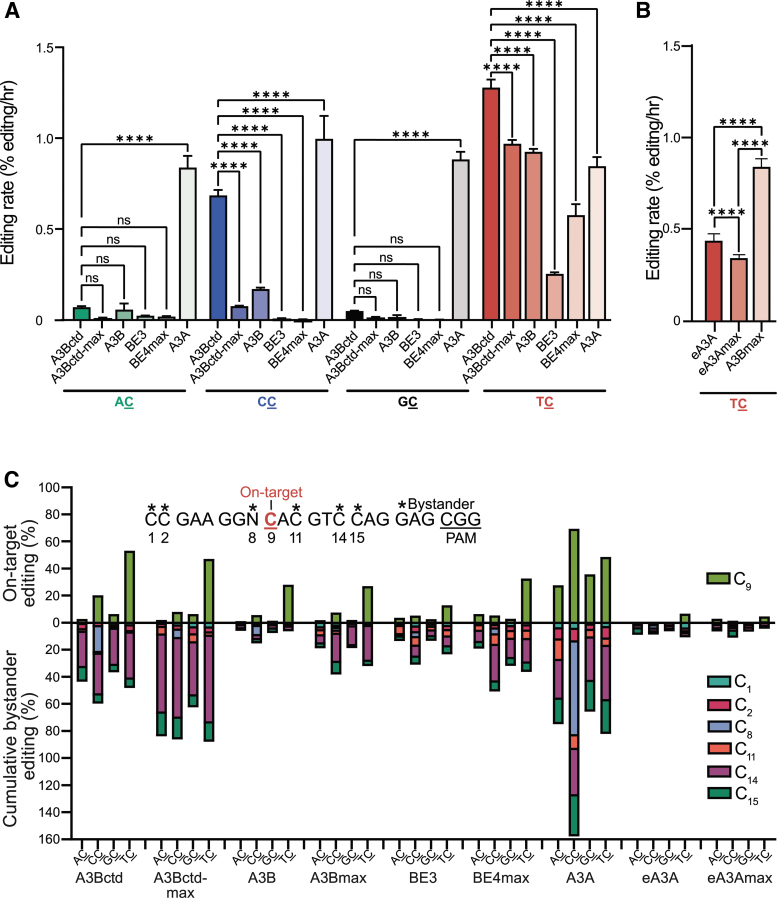
Comparisons of CBE editing rates, dinucleotide specificities, and bystander events. **(A, B)** Editing rate comparisons for the indicated CBEs with the four chromosomally integrated ARSENEL constructs after linear regression calculations (data are mean ± SD of biologically independent duplicate experiments, each with two technical replicates; ns = not significant; *p < 0.05; ****p < 0.0001 by two-way ANOVA). **(C)** Stacking bar graphs showing on-target (C_9_; above 0) and cumulative bystander (C_1_, C_2_, C_8_, C_11_, C_14_, C_15_; below 0) editing percentages calculated from direct Sanger sequencing analyses of PCR products from the indicated chromosomal ARSENEL editing reactions (each bar represents the mean of biologically independent duplicate experiments).

Next, we used the chromosomal ARSENEL system and editing rates to compare the A3Bctd CBE with established and novel CBEs to identify editing complexes with the highest on-target TC dinucleotide specificities and efficiencies (Fig. 5A). As above, A3Bctd CBE still exhibits the highest editing rate of 1.3% eGFP-positive edits/h with the TC reporter. The next best TC editing complexes cluster together with A3Bctd-max, full-length A3B, and A3A CBEs yielding similar eGFP-positivity rates of 1.0%, 0.9%, and 0.8% per hour. Surprisingly, given the recent reports,^19,52–54^ BE4max exhibits only 0.5% edits/h and BE3 ranks lowest among these complexes at 0.3% edits/h (see Supplementary Fig. S3A, B for supporting flow cytometry data and representative immunoblots).

As described above, A3Bctd CBE also edits CC at high rates (0.7% edits/h), which are approximately twofold lower than those for the TC reporter (Fig. 5A). Interestingly, A3Bctd-max CBE, which differs from the A3Bctd CBE through a codon-optimized Cas9n sequence, a longer linker, 2 Ugi's, and an N- and C-terminal NLS, exhibits ninefold lower CC reporter editing rates.^19^ This CBE also has significantly lower (almost negligible) rates of AC and GC editing. These results suggest that dinucleotide specificity ratios can be influenced by both the identity of the deaminase enzyme itself and by the composition of the gRNA-directed Cas9n-Ugi platform that is responsible for delivering the editing enzyme to the target DNA. This possibility is further supported by results with the A3A CBE, which efficiently (but nonspecifically) edits all four dinucleotide reporters (Fig. 5A and Supplementary Fig. S3A).

These results are likely due to A3A having a larger active site than A3Bctd and the highest documented rates of ssDNA C-to-U editing,^16,17,35,50,51^ as well as the A3A CBE having the same Cas9n-Ugi editing platform as the A3Bctd CBE.

Because A3Bctd-max has a lower CC editing rate than A3Bctd and the full-length A3B CBE intrinsically exhibits a high TC specificity (Fig. 5A and Supplementary Fig. S3A–C), we created a full-length A3Bmax CBE construct and compared it with the eA3A and eA3Amax CBE complexes, which are also considered to have a strong preference for TC dinucleotide substrates.^8,55,56^ Each of these three CBEs exhibits a clear preference for the TC reporter and minimal editing of the other three dinucleotide constructs (plate reader data in Fig. 5B and flow cytometry results in Supplementary Fig. S3C). The eA3A CBE has a higher editing rate compared with eA3Amax (Fig. 5B), which was a trend also evident in comparisons of A3Bctd and A3Bctd-max (Fig. 5A). However, full-length A3Bmax outperforms both eA3A and eA3Amax by approximately twofold in experiments with the TC dinucleotide reporter (Fig. 5B).

Next, we used Sanger sequencing chromatograms from high-fidelity PCR products to compare the repertoire of on-target versus bystander editing events in the gRNA binding region of eGFP. Bar graphs enable visual comparisons of C_9_ C-to-T editing for each CBE with data for each of the four reporters above the x-axis line, and cumulative bystander C-to-T editing events below the line, again, for each of the four reporters. Interestingly, of all TC-preferring editing complexes compared here, the full-length A3B CBE shows the highest overall ratio of on-target to bystander editing events (i.e., 28% on-target editing of TC_9_ and total of 6.2% bystander editing events in the TC reporter; Fig. 5C). Although other CBE complexes such as A3Bctd, A3Bctd-max, A3Bmax, BE4max, and BE3 also exhibit high C_9_ on-target editing capabilities, this desirable property is offset by much higher percentages of cumulative off-target editing events particularly for TC_14_ (Fig. 5C).

Interestingly, inserting full-length A3B into the BE4max backbone increased bystander editing at C_14_ compared with full-length A3B in the BE3 backbone. A3A CBE shows almost no specificity with similarly high rates of editing of C_9_ in all four dinucleotide contexts, and this liability is compounded by the highest levels of bystander editing events. On the opposite end of the spectrum, both eA3A and eA3Amax exhibit low rates of TC_9_ on-target editing and well as proportionately fewer bystander editing events. Additional sequencing data for A3Bctd-L7G, A3Bctd-Asp314-to-Glu, and AIDΔC CBEs are in Supplementary Figure S4.

### Characterization of CBE inhibitors using ARSENEL

Methods to systematically inhibit CBE activity may be desirable to prevent bystander and distil off-target events after the primary target has been edited successfully. The biological activity of A3 family members is virus restriction and, accordingly, viruses have evolved mechanisms to fight back by inhibiting A3 activity (reviewed by Ref.^57^). We recently discovered a mechanism of A3B inhibition in which the Epstein–Barr virus viral ribonucleotide reductase large subunit, BORF2, binds directly to the A3B active site and blocks catalytic activity.^38–40,58^

**FIG. 6.**
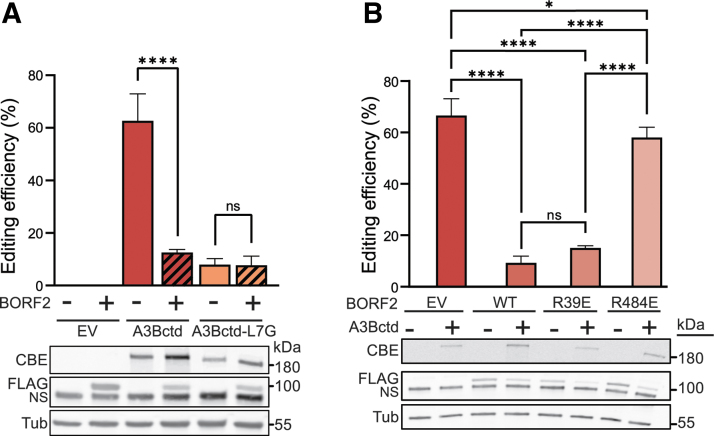
Demonstration of a virus-encoded inhibitor of A3Bctd CBE editing. **(A)** Efficiencies of A3Bctd CBE and A3Bctd-L7G in the absence and presence of BORF2 using flow cytometry to quantify editing of the chromosomally integrated TC reporter construct following CBE, gRNA, and BORF2 (or control) plasmid cotransfection into 293T cells (data are mean ± SD of biologically independent duplicate experiments, each with three technical replicates; ns = not significant; and ****p < 0.0001 by two-way ANOVA). The corresponding immunoblots from a representative experiment are shown below (the upper band in the anti-FLAG blot is BORF2-FLAG and the lower band is a cross-reacting cellular protein). **(B)** Efficiencies of A3Bctd CBE in the presence of the indicated BORF2 constructs using flow cytometry to quantify editing of the chromosomally integrated TC reporter construct following CBE, gRNA, and BORF2 (or control) plasmid cotransfection into 293T cells (data are mean ± SD of biologically independent triplicate experiments, each with two technical replicates; ns = not significant; *p < 0.05; ****p < 0.0001 by two-way ANOVA). The corresponding immunoblots from a representative experiment are shown below (the upper band in the anti-FLAG blot is BORF2-FLAG and the lower band is a cross-reacting cellular protein).

We therefore asked whether BORF2 is sufficiently potent to inhibit A3Bctd in the context of a powerful, gRNA-directed, nuclear-localizing CBE complex. Using the chromosomal TC reporter described above, we found that BORF2 inhibits editing by the A3Bctd CBE by 4.9-fold at 72 h post-transfection (Fig. 6A). Moreover, as the BORF2-A3Bctd interaction requires loop 7 residues,^39^ the A3Bctd-L7G CBE described above is resistant to BORF2 inhibition (Fig. 6A). The cryo-EM structure of the BORF2-A3Bctd complex and supporting biochemical and cellular studies show that BORF2-Arg484 is required for this direct interaction as well as for deaminase inhibition, but residues in the BORF2 dimerization interface such as Arg39 are dispensable.

In support of a direct inhibition mechanism, the single Arg484-to-Glu substitution prevents BORF2 from inhibiting chromosomal TC editing by the A3Bctd CBE, but an Arg39-to-Glu substitution has little effect (Fig. 6B). Thus, BORF2 dimerization is not required for A3Bctd CBE inhibition, which opens the door to future studies to try to leverage BORF2 monomers (or interacting fragments of the full polypeptide) to inhibit base editing.

## Discussion

Here we describe ARSENEL, which are live-cell fluorescence-based assays for quantifying the dinucleotide editing specificities and efficiencies of CBEs. ARSENEL can be used for assessing individual editing complexes and for testing candidate inhibitors, as exemplified here for the EBV protein BORF2. ARSENEL may also be useful for future studies using medium- to high-throughput screens to identify new CBEs with enhanced properties. ARSENEL is the first system to our knowledge to enable direct isogenic comparisons of CBE dinucleotide editing efficiencies, although other fluorescent base editing reporter systems such as BE-FLARE and TREE may be adapted for this purpose (as described in the last paragraph of Discussion). ARSENEL also has added value by enabling the simultaneous assessment of on-target and bystander editing events within the gRNA binding region by Sanger sequencing, as in studies here, or deep sequencing of high-fidelity PCR products.

An important observation from experiments with ARSENEL is that the wild-type full-length A3B CBE exhibits the highest overall specificity for TC substrates (although not the highest efficiency). This is best evidenced by comparing the percentage of exclusive on-target C_9_ chromosomal editing events (28%) versus the sum of the percentage of all bystander editing events (6.2%) (Fig. 5C). This CBE also has relatively low frequencies of editing the three different non-TC dinucleotides (Fig. 5A–C). In comparison, although other CBEs have an equally strong (BE4max) or a weaker preference (other constructs) for editing the on-target TC_9_ dinucleotide motif, all of these competing CBEs cause higher frequencies of adjacent off-target editing events (Fig. 5A–C).

These results illustrate the tremendous challenge of identifying a CBE with a high on-target efficiency and no liabilities. A precise molecular explanation of the desirable attributes of the full-length A3B CBE will require high-resolution structural studies but is likely to involve a role for the noncatalytic N-terminal domain (A3Bntd), which is unique to this construct. In comparison, the A3A CBE lacks specificity as evidenced by high-frequency editing of all four ARSENEL dinucleotide substrates (Fig. 5A, C, and Supplementary Fig. S3A). The A3A CBE also exhibits the highest levels of bystander editing events (Fig. 5C). This editing construct is therefore the most active and promiscuous of all CBEs tested here, which could be useful in mutagenesis studies where many different mutations are desirable and phenotypic screens can be leveraged to help identify useful mutants.

A notable curiosity from studies here with ARSENEL is that all CBEs are not equal in producing transversion mutation outcomes. If both uracil excision by UNG2 (despite all constructs including at least one Ugi) and translesion synthesis by REV1 are stochastic events, then all CBEs should yield transversion mutation outcomes at rates proportional to their overall editing and C-to-T transition mutation capabilities. This is not likely to be the case because, for example, the AIDΔC CBE appears to produce a C-to-A transversion bias at its preferred dinucleotide target in the GC reporter (Fig. 3E). This outcome was also noted in recent literature where AID-BE4max was reported to have a low, but significant frequency of C-to-A and C-to-G edits.^54^

A provocative interpretation of this result is that AID (or an AID-associated factor) may somehow have the capacity to influence downstream mutagenic DNA repair outcomes (reviewed by Refs.^59,60^). This property may be intrinsic to AID and advantageous for its physiological function in somatic hypermutation and class switch recombination, and it may be harnessed for use as a C-to-A transversion base editor.^61^

A limitation of the studies here is that we focus on a single gRNA binding site in eGFP to isogenically compare editing of the four different dinucleotide contexts by a panel of CBEs. It is possible that other reporters and/or chromosomal sites will yield different results. In addition, at least for TC preferring editors, ARSENEL may underestimate efficiencies due to bystander C-to-T editing at C_15_, which introduces a stop codon. Another limitation of the ARSENEL system is nucleobase constraints at the −2, +1, and +2 positions relative to the target cytosine, necessarily G, A, and C here to maintain eGFP reporter functionality (Fig. 1A and Supplementary Fig. S1A, B). Although less important than the −1 position, these up- and downstream nucleobases make additional contributions to APOBEC-ssDNA interactions and overall cytosine deamination substrate preferences.^27–29,31–35,44,45,48,51,62–67^

This could explain why here, for instance, the AIDΔC and A3B-L7G CBE constructs exhibit lower than expected editing rates (ARSENEL editing motifs are subideal for AID and A3G, which prefer WRC and CCC, respectively). Future efforts, and particularly those aiming to develop therapeutic constructs (ideally with the gRNA and CBE encoded by the same construct for additional consistency), may want to design custom editors that are as-specific-as-possible for the tetra- or pentanucleotide context of the disease mutation requiring correction.

Previous studies including our own have used fluorescence to measure the editing activity of CBEs.^17,36,51,68–72^ BE-FLARE^69^ and TREE^72^ both utilize the same target site that through a single C-to-T mutation converts blue fluorescent protein (BFP) fluorescence to GFP via a His66 to Tyr66 amino acid change. Because the upstream Thr65 codon in BFP can be wobbled, either of these systems could be expanded in future studies to examine dinucleotide editing preferences as we have done here for ARSENEL (and enable the examination of a different repertoire of bystander cytosine editing events relative to the PAM site; e.g., C_2_, C_4_, C_5_, C_13_, and C_18_ for BE-FLARE). In comparison, GO^71^ is a gain-of-signal system that restores GFP function, but it is restricted to editing of a phenotypically constrained AC dinucleotide. In contrast, BEAR^70^ utilizes a split GFP system that, upon editing of an aberrant splice site, restores splicing and fluorescence. However, a potential drawback to this system is that single nucleobase changes upstream of the target C are likely to alter splicing and therefore reporter efficiencies, which may complicate experiments to assess broader CBE editing preferences. DNA sequencing, including both Sanger sequencing and next-generation sequencing, can then be used to further assess specificity and gain insights into bystander rates.

## Conclusions

The ARSENEL system described here is unique in that it is the first to enable a systematic comparison of dinucleotide editing preferences of virtually any CBE. In addition, we found that a full-length A3B CBE has the best overall on-target TC dinucleotide specificity, whereas an A3A CBE shows higher editing rates but also high promiscuity with loose dinucleotide specificities and high levels of bystander editing. In addition to enabling direct CBE comparisons, the ARSENEL system is also useful for real-time characterization of natural and synthetic APOBEC inhibitors. Future studies may be able to leverage the ARSENEL system to help develop therapeutic CBEs with maximal specificity and efficiency for each individual disease target.

## Supplementary Material

Supplemental data

Supplemental data

Supplemental data

Supplemental data

Supplemental data

## Data Availability

The data underlying this article are available in the article and in its online Supplementary Material. All ARSENEL reporter and novel base editor constructs have been provided to Addgene for distribution to the scientific community. EditR can be accessed at https://moriaritylab.shinyapps.io/editr_v10.
