# Supplementary material for: APOBEC Reporter Systems for Evaluating diNucleotide Editing Levels
Source: CRISPR J. 2023 Oct 10;6(5):430–46. doi: 10.1089/crispr.2023.0027 (PMC10611974; doi:10.1089/crispr.2023.0027)
Supplement: Supplemental data [file Suppl_TableS1.pdf]

Supplementary Table S1. Oligonucleotides used in this study.

Y93 AC SDM Forward	GCCATGCCCGAAGGacACGTCCAGGAGCGgACCAT CTTCTTCAA
Y93 AC SDM Reverse	TTGAAGAAGATGGTcCGCTCCTGGACGTgtCCTTCG GGCATGGC
Y93 CC SDM Forward	GCCATGCCCGAAGGccACGTCCAGGAGCGgACCAT CTTCTTCAA
Y93 CC SDM Reverse	TTGAAGAAGATGGTcCGCTCCTGGACGTggCCTTC GGGCATGGC
Y93 GC SDM Forward	GCCATGCCCGAAGGgcACGTCCAGGAGCGgACCAT CTTCTTCAA
Y93 GC SDM Reverse	TTGAAGAAGATGGTcCGCTCCTGGACGTgcCCTTC GGGCATGGC
GFP Sequencing Forward	CACCATCGTGGAACAGTACGAAC
GFP Y93 AC gRNA Forward	caccgCCGAAGGacACGTCCAGGAG
GFP Y93 AC gRNA Reverse	gaacCTCCTGGACGTgtCCTTCGGc
GFP Y93 CC gRNA Forward	caccgCCGAAGGccACGTCCAGGAG
GFP Y93 CC gRNA Reverse	gaacCTCCTGGACGTggCCTTCGGc
GFP Y93 GC gRNA Forward	caccgCCGAAGGgcACGTCCAGGAG
GFP Y93 GC gRNA Reverse	gaacCTCCTGGACGTgcCCTTCGGc
GFP Y93 TC gRNA Forward	caccgCCGAAGGtcACGTCCAGGAG
GFP Y93 TC gRNA Reverse	gaacCTCCTGGACGTgaCCTTCGGc
gRNA Sequencing Forward	TTTCTTGGGTAGTTTGCAGTTTT
A3Bctd-L7G Cloning Forward	CGGTGCCACTTTTTCAAGTT
A3Bctd-L7G Cloning Reverse	TGAGGTCCCGGGAGTCTCGCTGCCGCTGTTTCCC TGATTCTGG AGAATGGCC
A3Bctd-D314E SDM Forward	GCCCGCATCTATGAgTACGACCCCCTA
A3Bctd-D314E SDM Reverse	TAGGGGGTCGTAcTCATAGATGCGGGC
A3A N57G SDM Forward	AGGGGCTTTCTACACGGCCAGGTGAGTCCAGGA
A3A N57G SDM Forward	TCCTGGACTCACCTGGCCGTGTAGAAAGCCCCT
BE4max Inverse PCR	TGATCGGCAACAAGCCAGAGT
BE4max Inverse PCR Reverse	CTTCTTCTTTGGTGACTCGAACTCGCTTC
BE4max Esp3I Cloning Forward	CACCAAAGAAGAAGCGGAAAGTCGAGACGATTTAA TGCGTCTCTTCTGGAGGATCTAGCGGAG
BE4max Esp3I Cloning Reverse	TTGTTGCCGATCACTTCCTCCACCTCCTCA
A3Bmax Cloning Forward	aaaaaacgtctcccagaGTTTCCCTGATTCTGGAGAATG
A3Bctd-max Cloning Forward	ttttttcgtctcaaagtcGATCCAGACACATTCACTTTCAAC
A3Bctd-max Cloning Reverse	aaaaaacgtctcccagaGTTTCCCTGATTCTGGAGAATG
eA3Amax Cloning Forward	ttttttcgtctcaaagtcGAAGCCAGCCCAGCA
eA3Amax Cloning Reverse	aaaaaacgtctcccagaTCCGTTTCCCTGATTCTGG
gDNA GFP PCR Amplification Forward	TCTTCTAACATGCGGTGACG
gDNA GFP PCR Amplification Reverse	GAACTCCAGCAGGACCATGT
gDNA GFP Sequencing Forward	CCTGAAGTTCATCTGCACCA
gDNA GFP Sequencing Reverse	CACRCTRCCRTCCTC
