# Supplementary material for: APOBEC Reporter Systems for Evaluating diNucleotide Editing Levels
Source: CRISPR J. 2023 Oct 10;6(5):430–46. doi: 10.1089/crispr.2023.0027 (PMC10611974; doi:10.1089/crispr.2023.0027)
Supplement: Supplemental data [file Suppl_FigureS1.pdf]

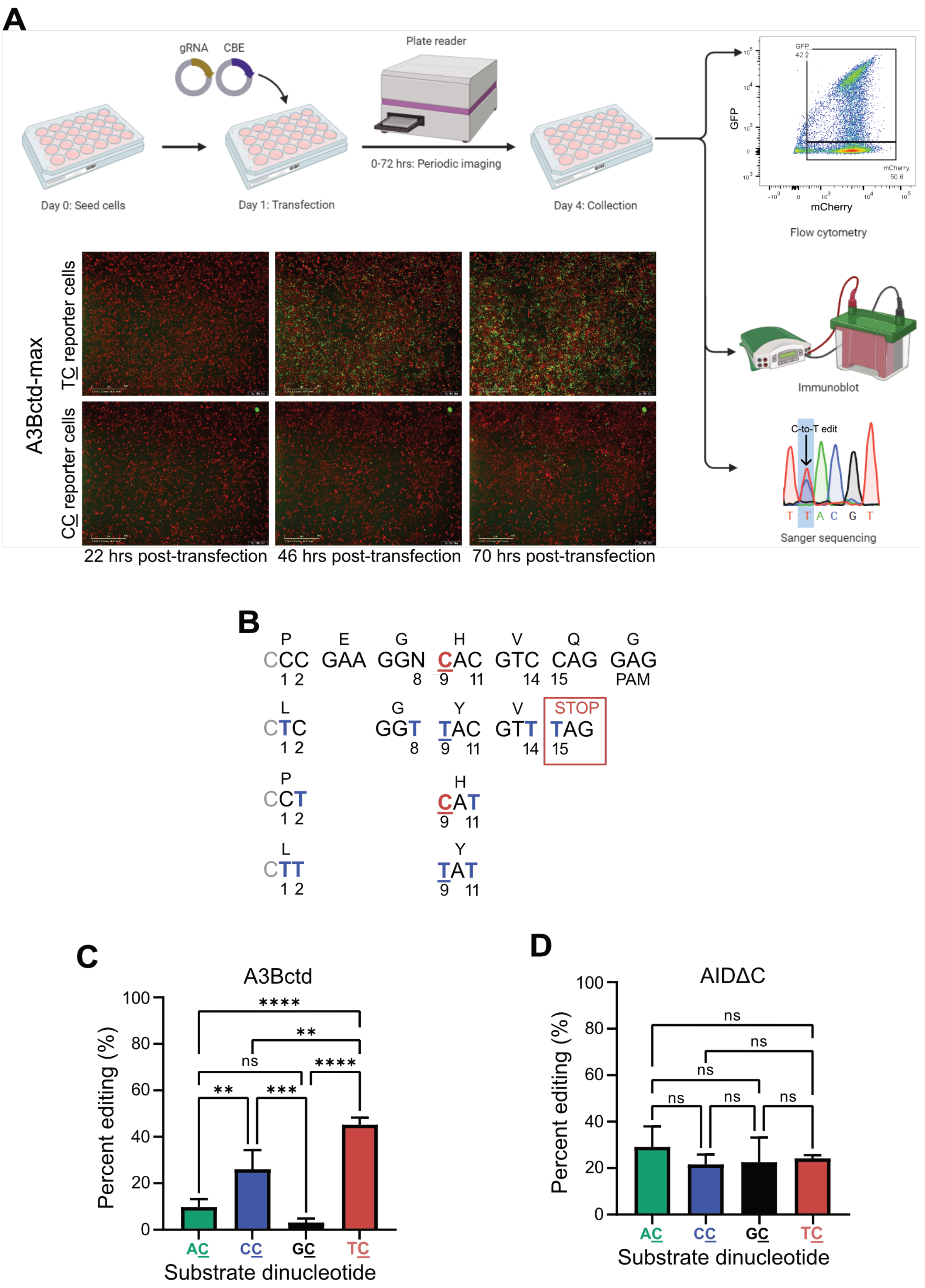
A
gRNA
CBE
Plate reader
Day 0: Seed cells
Day 1: Transfection
0-72 hrs: Periodic imaging
Day 4: Collection
GFP
42.2
mCherry
50.6
Flow cytometry
Immunoblot
C-to-T edit
T T A C G T
Sanger sequencing
A3Bctd-max
TC reporter cells
CC reporter cells
22 hrs post-transfection
46 hrs post-transfection
70 hrs post-transfection
B
P E G H V Q G
CCC GAA GGN CAC GTC CAG GAG
1 2 8 9 11 14 15 PAM
L G Y V STOP
CTC GGT TAC GTT TAG
1 2 8 9 11 14 15
P H
CCT CAT
1 2 9 11
L Y
CTT TAT
1 2 9 11
C
A3Bctd
Percent editing (%)
100
80
60
40
20
0
****
**
ns
****
**
***
AC CC GC TC
Substrate dinucleotide
D
AIDΔC
Percent editing (%)
100
80
60
40
20
0
ns
ns
ns
ns
ns
ns
AC CC GC TC
Substrate dinucleotide

Supplementary Figure S1. Assay workflow and episomal editing by A3Bctd and AIDΔC CBEs.

(**A**) General workflow for ARSENEL assay. For episomal editing experiments, 293T cells are seeded at an appropriate density in plates (day 0), co-transfected with ARSENEL reporter (not shown), CBE, and gRNA plasmids (day 1), and quantified at multiple timepoints (day 1-4) by fluorescent imaging (Cytation or Incucyte instruments) or flow cytometry. Chromosomal editing experiments are done similarly except 293T cells pools are pre-engineered by transduction and drug selection to harbor one of the four dinucleotide reporters. Additional analyses such as immunoblots for CBE expression and Sanger sequencing for editing within the gRNA-targeted region of eGFP are done using cells from parallel reactions 48-72 hrs post-transfection. Representative plate images, flow cytometry dot plot, and Sanger sequencing chromatograms are shown as examples.

(**B**) List of eGFP amino acid changes that can arise from editing the indicated cytosines in the ARSENEL system. The target C_9 is labeled in red and underlined, and C-to-T mutations are blue.

(**C-D**) Efficiencies of A3Bctd and AIDΔC CBEs, respectively, using flow cytometry to quantify editing of the four chromosomally integrated ARSENEL constructs 48 hrs post-transfection of CBE and gRNA plasmids into 293T cells [(eGFP+ / mCherry+) x 100; each histogram bar is the mean +/- SD of three biologically independent experiments]. These flow cytometry results are an independent quantification of experiments shown in Figure 1B and 1C. Significance is based on an ordinary one-way ANOVA (ns = not significant; ** = $p < 0.01$; *** = $p < 0.001$; **** = $p < 0.0001$).
