# Supplementary material for: APOBEC Reporter Systems for Evaluating diNucleotide Editing Levels
Source: CRISPR J. 2023 Oct 10;6(5):430–46. doi: 10.1089/crispr.2023.0027 (PMC10611974; doi:10.1089/crispr.2023.0027)
Supplement: Supplemental data [file Suppl_FigureS2.pdf]

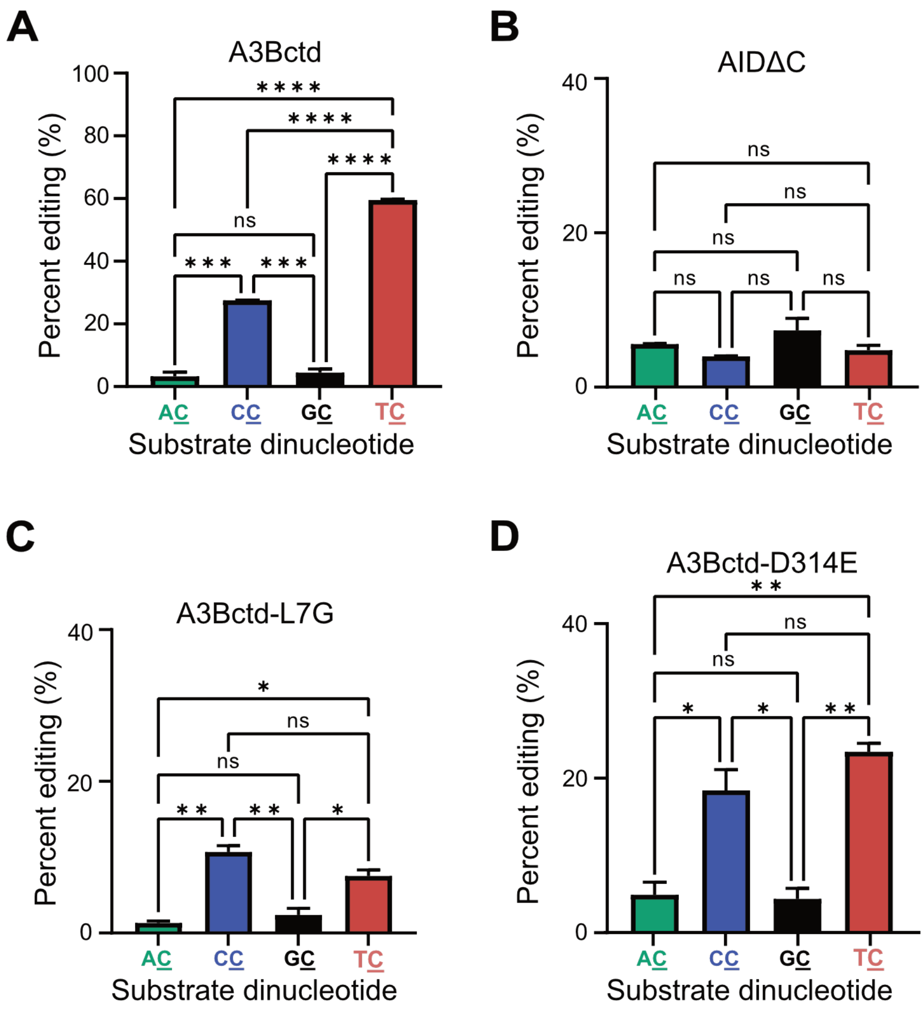

Supplementary Figure S2. Chromosomal editing by A3Bctd, AIDΔC, A3Bctd-L7G, and A3Bctd-D314E.

(**A-D**) Chromosomal dinucleotide base editing frequencies of A3Bctd, AIDΔC, A3Bctd-L7G, and A3Bctd-D314E CBEs, respectively, as quantified 72 hrs post-transfection by flow cytometry [(eGFP+ / mCherry+) x 100; each histogram bar is the mean +/- SD of biologically independent duplicate experiments each with two technical replicates]. These flow cytometry results are an independent quantification of experiments run in parallel to those shown in Figures 2A, 2C, and 4B-C. Significance is based on an ordinary one-way ANOVA (ns = not significant; * = $p < 0.05$; ** = $p < 0.01$; *** = $p < 0.001$; **** = $p < 0.0001$).
