# Supplementary material for: APOBEC Reporter Systems for Evaluating diNucleotide Editing Levels
Source: CRISPR J. 2023 Oct 10;6(5):430–46. doi: 10.1089/crispr.2023.0027 (PMC10611974; doi:10.1089/crispr.2023.0027)
Supplement: Supplemental data [file Suppl_FigureS3.pdf]

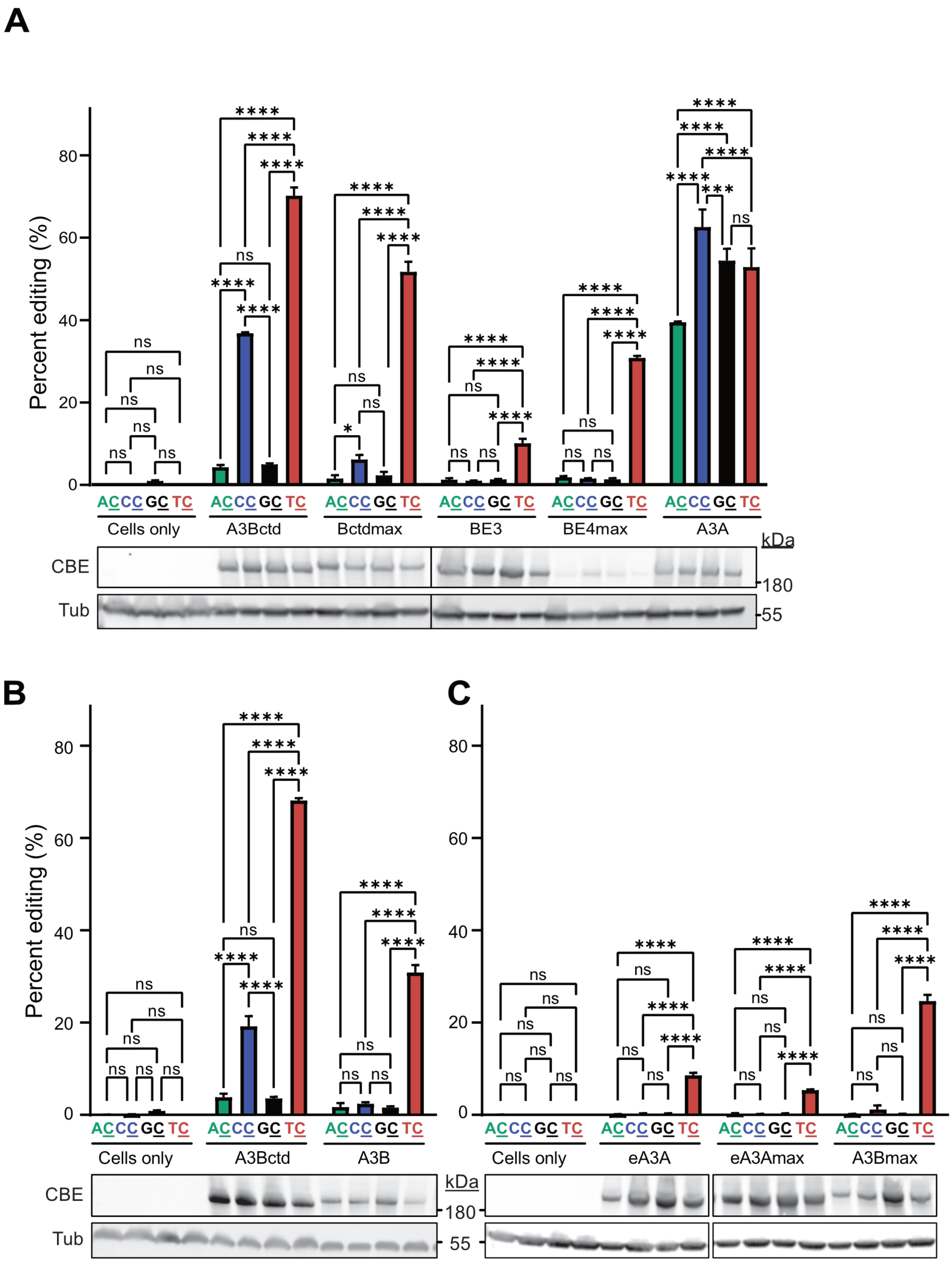
A
Percent editing (%)
0
20
40
60
80
ACCC GC TC
Cells only
A3Bctd
Bctdmax
BE3
BE4max
A3A
kDa
CBE
Tub
180
55
B
Percent editing (%)
Cells only
A3Bctd
A3B
C
Cells only
eA3A
eA3Amax
A3Bmax

Supplementary Figure S3. Comparisons of chromosomal editing by different CBEs.

(**A**) Chromosomal dinucleotide base editing frequencies of A3Bctd, A3Bctd-max, BE3, BE4max, and A3A 60 hrs post-transfection by flow cytometry [(eGFP+ / mCherry+) x 100; each histogram bar is the mean +/- SD of biologically independent duplicate experiments each with two technical replicates]. These flow cytometry results are an independent quantification of reactions run in parallel to those shown in Figure 5A. Significance is based on a 2way ANOVA (ns = not significant; * = p < 0.05; ** = p < 0.01; *** = p < 0.001; **** = p < 0.0001). Representative immunoblots are depicted below with bands positioned below their respective bar (these images are from the same blot, and split for presentation due to gel smiling).

(**B**) Chromosomal dinucleotide base editing frequencies of A3Bctd and full-length A3B 60 hrs post-transfection by flow cytometry [(eGFP+ / mCherry+) x 100; each histogram bar is the mean +/- SD of biologically independent duplicate experiments each with two technical replicates]. These flow cytometry results are an independent quantification of reactions run in parallel to those shown in Figure 5A. Significance is based on a 2way ANOVA (ns = not significant; * = p < 0.05; ** = p < 0.01; *** = p < 0.001; **** = p < 0.0001). Representative immunoblots are depicted below with bands positioned below their respective bar.

(**C**) Chromosomal dinucleotide base editing frequencies of A3Bmax, eA3A, and eA3Amax 60 hrs post-transfection by flow cytometry [(eGFP+ / mCherry+) x 100; each histogram bar is the mean +/- SD of biologically independent duplicate experiments each with two technical replicates]. These flow cytometry results are an independent quantification of reactions run in parallel to those shown in Figure 5B. Significance is based on a 2way ANOVA (ns = not significant; * = p < 0.05; ** = p < 0.01; *** = p < 0.001; **** = p < 0.0001). Representative immunoblots are depicted below with bands positioned below their respective bar (these images are from the same blot, and split for presentation to remove non-relevant data between eA3A and eA3Amax result).
