# Supplementary material for: APOBEC Reporter Systems for Evaluating diNucleotide Editing Levels
Source: CRISPR J. 2023 Oct 10;6(5):430–46. doi: 10.1089/crispr.2023.0027 (PMC10611974; doi:10.1089/crispr.2023.0027)
Supplement: Supplemental data [file Suppl_FigureS4.pdf]

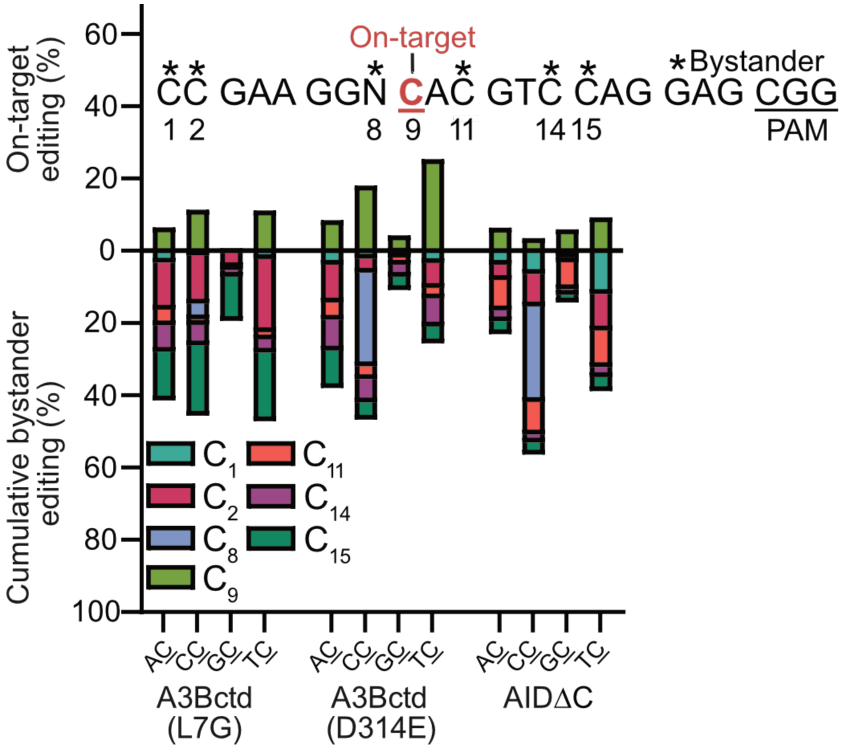

Supplementary Figure S4. Comparison of on-target and bystander events.

Stacking bar graphs showing on-target (C9; above 0) and cumulative bystander (C1, C2, C8, C11, C14, C15; below 0) editing percentages calculated from Sanger sequencing analyses of PCR products from the indicated chromosomal ARSENEL editing reactions (each bar represents the mean of biologically independent duplicate experiments).
